# Cox-2 Antagonizes the Protective Effect of Sevoflurane on Hypoxia/Reoxygenation-Induced Cardiomyocyte Apoptosis through Inhibiting the Akt Pathway

**DOI:** 10.1155/2021/4114593

**Published:** 2021-12-07

**Authors:** Chunyan Guo, Lei Zhang, Yaoxing Gao, Junzhi Sun, Lingling Fan, Yuguang Bai, Jing Zhang, Gaowa Naren, Jiwen Yang, Libiao Li

**Affiliations:** ^1^Department of Anesthesiology, The Affiliated Hospital of Inner Mongolia Medical University, Hohhot, China; ^2^Department of Ultrasound, The Affiliated Hospital of Inner Mongolia Medical University, Hohhot, China; ^3^Department of Stomatology, Tongliao Hospital, Tongliao, China; ^4^Department of Neurosurgery, The Affiliated Hospital of Inner Mongolia Medical University, Hohhot, China

## Abstract

**Objective:**

To uncover the protective role of sevoflurane on hypoxia/reoxygenation-induced cardiomyocyte apoptosis through the protein kinase B (Akt) pathway.

**Methods:**

An *in vitro* hypoxia/reoxygenation (H/R) model was established in cardiomyocyte cell line H9c2. Sevoflurane (SEV) was administrated in H9c2 cells during the reoxygenation period. Viability, layered double hydroxide (LDH) release, and apoptosis in H9c2 cells were determined to assess H/R-induced cell damage. Relative levels of apoptosis-associated genes were examined. Moreover, phosphorylation of Akt was determined.

**Results:**

H/R injury declined viability and enhanced LDH release and apoptotic rate in H9c2 cells. Cyclooxygenase-2 (Cox-2) was upregulated following H/R injury, which was partially reversed by SEV treatment. In addition, SEV treatment reversed changes in viability and LDH release owing to H/R injury in H9c2 cells, which were further aggravated by overexpression of Cox-2. The Akt pathway was inhibited in H9c2 cells overexpressing Cox-2.

**Conclusions:**

Sevoflurane protects cardiomyocyte damage following H/R *via* the Akt pathway, and its protective effect was abolished by overexpression of Cox-2.

## 1. Introduction

So far, ischemic heart diseases are the leading fatal cardiovascular diseases globally [1]. Myocardial ischemia/reperfusion (I/R) leads to apoptosis and necrosis of cardiomyocytes, further aggravating cardiac insufficiency, ventricular remodeling, and even heart failure [2]. Inhibition of apoptosis attributes to alleviate I/R-induced myocardial lesions, cardiomyocyte loss, and ventricular contractile dysfunction [3]. Severe hypoxia is the most typical feature of myocardial ischemia, which eventually results in ROS accumulation, intracellular calcium overload, inflammatory response, energy deficiency, and other serious lesions. Notably, reoxygenation following hypoxia may also cause secondary damage, eventually leading to cardiomyocyte death [4, 5]. It is of significance to clarify mechanisms of H/R injuries.

Protein kinase B (Akt) is a serine/threonine-specific protein kinase. Akt is extensively involved in cellular processes such as glucose metabolism, gene transcription, and cell behaviors [6, 7]. Accumulating evidences have suggested that the activated PI3K/Akt pathway following myocardial I/R injury induces a cardioprotective function [8, 9]. Cyclooxygenase-2 (Cox-2) is a cyclooxygenase isoform catalyzing the conversion of arachidonic acid to prostaglandins [10]. During the process of myocardial ischemia, Cox-2 is upregulated in myocardium [11, 12]. Besides, Cox-2 level is associated with the severity of apoptosis in myocardial infarction [13].

Sevoflurane (SEV) is an anesthetic that is widely applied in cardiac surgeries. Compared with other anesthetics, SEV has the advantages of short induction time, short recovery time, and high safety [14]. Recent studies have shown that SEV pretreatment/posttreatment has significant protective effects on myocardial H/R injury [15, 16]. In this paper, H9c2 cells were utilized for constructing an *in vitro* H/R model. We mainly explored the potential protective role of SEV in myocardial H/R injury and the involvement of Cox-2.

## 2. Materials and Methods

### 2.1. Cell Culture

Rat embryonic cardiomyocyte cell line H9c2 was provided by Cell Bank (Shanghai, China). Cells were cultured in Dulbecco's modified eagle medium (DMEM) (Gibco, Rockville, MD, USA) containing 10% fetal bovine serum (FBS) (Gibco, Rockville, MD, USA), 100 *μ*g/mL penicillin, and 100 mg/mL streptomycin. Fresh medium was replaced every 2-3 days. Cell passage was performed at 80-90% confluence.

### 2.2. Construction of In Vitro H/R Model

H9c2 cells were cultured in low-serum medium (0.5% FBS) and exposed to 95% N_2_/5% CO_2_. After 2 h hypoxic culture, cells were cultured in fresh medium (10% FBS) and exposed to 95% air/5% CO_2_. After reoxygenation for 1 h, the *in vitro* H/R model was constructed. Cells in the NC group were routinely cultured. SEV was administrated in cells of the H/R + SEV group during reoxygenation period.

### 2.3. Transfection

pcDNA3.1-Cox-2 (Cox-2 OE) (NM_017232.3) was constructed. Cells were transfected using Lipofectamine 2000 (Thermo Fisher Scientific, Waltham, MA, USA) as previously reported.

### 2.4. Cell Counting Kit-8 (CCK-8)

Cells were inoculated in a 96-well plate. 100 *μ*L of 10% CCK-8 was applied in each well. At the appointed time points, absorbance value at 450 nm of each sample was recorded using the CCK-8 kit (Dojindo Laboratories, Kumamoto, Japan) for plotting the viability curves.

### 2.5. Layered Double Hydroxide (LDH) Release Determination

Cell supernatant was collected and incubated with 60 *μ*L of LDH working solution. After 30 min incubation in dark at room temperature on an oscillator, an absorbance value at 490 nm was recorded.

### 2.6. Flow Cytometry

Cells were collected, washed in precold PBS twice, and resuspended in 500 *μ*L of binding buffer containing 5 *μ*L of annexin V-FITC (fluorescein isothiocyanate) and 5 *μ*L of propidium iodide (PI) in dark. 30 min later, cell apoptosis was determined by flow cytometry at 488 nm excitation and 600 nm emission.

### 2.7. Quantitative Real-Time Polymerase Chain Reaction (qRT-PCR)

TRIzol method (Invitrogen, Carlsbad, CA, USA) was applied for isolating cellular RNA. Through reverse transcription of RNA, the extracted complementary deoxyribose nucleic acid (cDNA) was used for PCR detection using the DBI Bestar SybrGreen qPCR Master Mix (DBI Bioscience, Shanghai, China) on Stratagene Mx3000P Real-Time PCR system (Agilent Technologies, Santa Clara, CA, USA). Glyceraldheyde 3-phosphate dehydrogenase (GAPDH) was used as the internal reference. Cox-2: 5′-ATTGCTGGCCGGGTTGCTGG-3′ (F), 5′-TCAGGGAGAAGCGTTTGCGGT-3′ (R); GAPDH: 5′-TCCCTCAAGATTGTCAGCAA-3′ (F), 5′-AGATCCACAACGGATACATT-3′ (R).

### 2.8. Western Blot

Cells were lysed for isolating cellular protein and electrophoresed. Protein samples were loaded on polyvinylidene fluoride (PVDF) membranes (Millipore, Billerica, MA, USA). Subsequently, nonspecific antigens were blocked in 5% skim milk for 2 hours. Gapdh (60004-1-Ig), Cox-2 (66351-3-Ig), Akt (60203-2-Ig), and p-Akt (66444-1-Ig) were all purchased from Proteintech. Membranes were reacted with primary and secondary antibodies for the indicated time. Band exposure and analyses were finally conducted.

### 2.9. Statistical Analysis

Statistical Product and Service Solutions (SPSS) 22.0 (IBM, Armonk, NY, USA) was used for all statistical analysis. Data were expressed as mean ± SD (standard deviation). Comparison between multiple groups was done using one-way ANOVA test followed by post hoc test (Least Significant Difference). *p* < 0.05 indicated the significant difference.

## 3. Results

### 3.1. SEV Protected Cardiomyocyte Apoptosis following H/R

**Figure 1:**
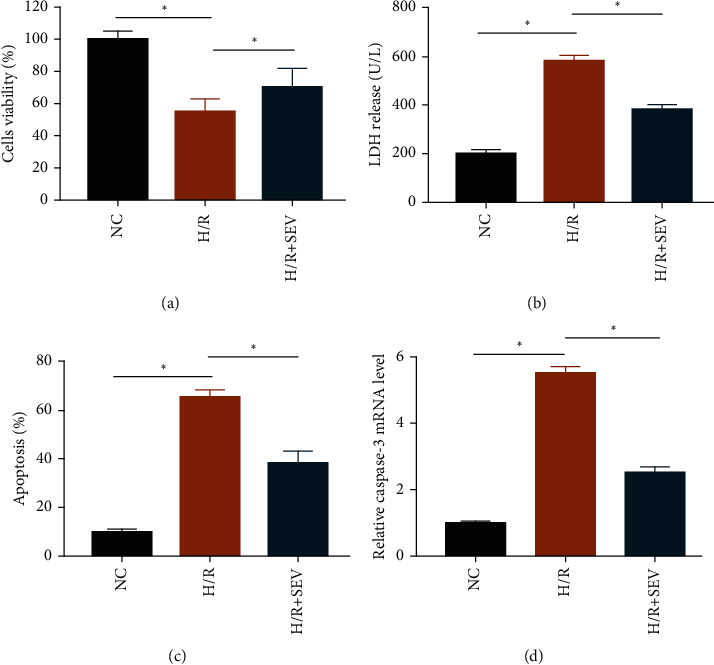
SEV protected H/R-induced cardiomyocyte apoptosis. H9c2 cells were assigned into the NC group, H/R group, and H/R + SEV group. Cell viability (a), LDH release (b), apoptotic rate (c), and mRNA level of caspase-3 (d).

After establishing the H/R model in H9c2 cells, viability markedly decreased (Figure 1(a)), while LDH release (Figure 1(b)) and apoptosis increased (Figure 1(c)). Notably, SEV treatment partially reversed the above trends. Consistently, caspase-3 was markedly upregulated in the H/R group, and the increased level of caspase-3 was reduced by SEV (Figure 1(d)). Therefore, SEV markedly alleviated cardiomyocyte apoptosis following H/R injury.

### 3.2. Cox-2 Was Upregulated following H/R

**Figure 2:**
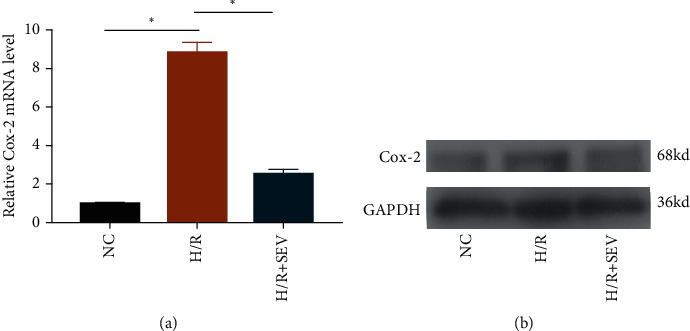
Cox-2 was upregulated following H/R. H9c2 cells were assigned into the NC group, H/R group, and H/R + SEV group. Relative mRNA (a) and protein level (b) of Cox-2.

QRT-PCR data showed that Cox-2 was upregulated at post-H/R in cardiomyocytes, and SEV treatment could partially relieve this increased trend (Figure 2(a)). Similarly, protein level changes of Cox-2 presented the same trends as its mRNA level (Figure 2(b)).

### 3.3. Overexpression of Cox-2 Suppressed Cardioprotective Role of SEV

**Figure 3:**
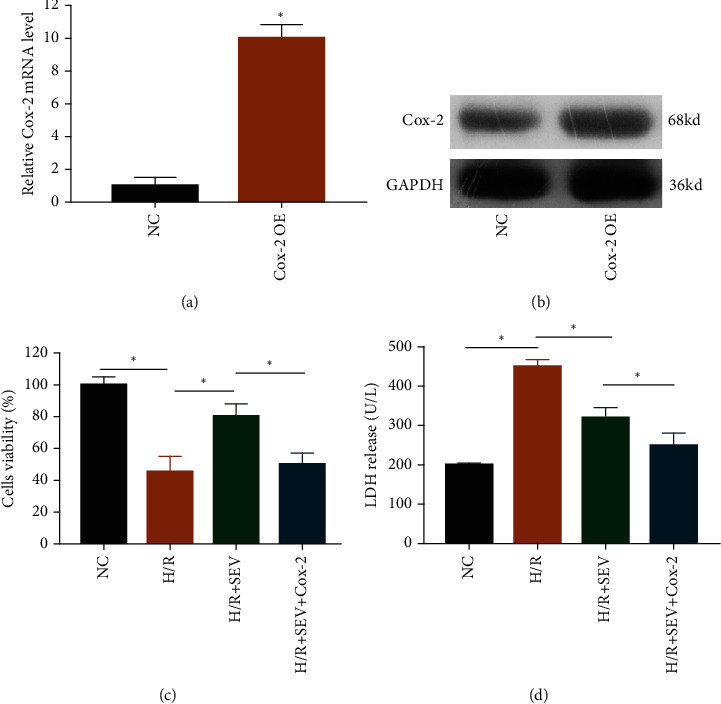
Overexpression of Cox-2 suppressed cardioprotective role of SEV. The mRNA (a) and protein level (b) of Cox-2 in H9c2 cells transfected with NC or Cox-2 OE. Cell viability (c) and LDH release (d) in H9c2 cells of NC group, H/R group, H/R + SEV group, and H/R + SEV + Cox-2 group.

To uncover the potential involvement of Cox-2 in cardioprotective role of SEV following H/R, pcDNA3.1-Cox-2 (Cox-2 OE) was constructed. Its transfection efficacy was firstly verified in H9c2 cells (Figures 3(a) and 3(b)). Interestingly, protective effects of SEV on viability enhancement (Figure 3(c)) and LDH release inhibition (Figure 3(d)) in H/R-induced cardiomyocytes were partially abolished by overexpression of Cox-2. Hence, the protective property of SEV on cardiomyocytes following H/R was largely limited by Cox-2.

### 3.4. Overexpression of Cox-2 Inhibited SEV-Induced p-Akt Upregulation

**Figure 4:**
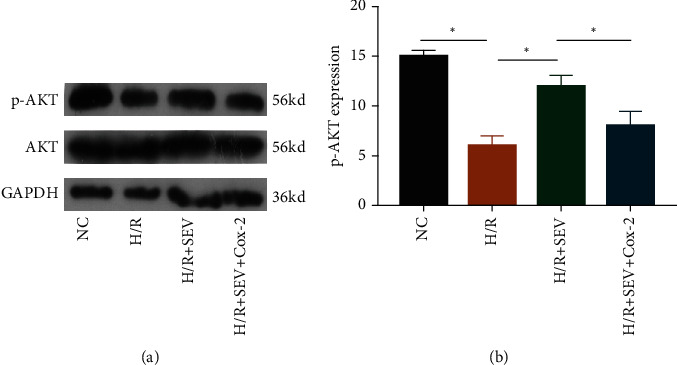
Overexpression of Cox-2 inhibited SEV-induced p-Akt upregulation. Protein levels of p-Akt and Akt in H9c2 cells of NC group, H/R group, H/R + SEV group, and H/R + SEV + Cox-2 group.

Accumulating evidences have proven the critical function of Akt in cardiomyocyte survival [17]. Here, p-Akt was downregulated following H/R injury. SEV treatment markedly increased protein level of p-Akt, which was inhibited by Cox-2 overexpression (Figures 4(a) and 4(b)). The Akt pathway was responsible for cardioprotective effect of SEV.

## 4. Discussion

Ischemic heart diseases pose extremely high morbidity and mortality. Blood flow reperfusion and reoxygenation following ischemic injury are the conventional treatments. However, they may result in I/R and H/R injuries [18]. During the pathological progressions of I/R and H/R, a series of complicated events including oxidative stress, apoptosis, and inflammation significantly affect therapeutic efficacy [19, 20]. Prevention and treatment of I/R and H/R have been well concerned.

SEV is a commonly used inhalation anesthetic with multiple advantages. Animal experiments have proven that SEV is able to alleviate myocardial I/R injury in rats [21–23]. Nevertheless, specific mechanisms underlying the protective role of SEV in I/R remain unclear. Our findings firstly showed that declined viability, enhanced LDH release, and apoptosis in H9c2 cells undergoing H/R, suggesting the successful construction of an *in vitro* H/R model. Notably, SEV administration during reoxygenation greatly protected H/R-induced cardiomyocyte injuries.

Akt is a vital kinase involved in cell survival [24]. Acute activation of Akt markedly protects apoptosis and necrosis of cardiomyocytes under external stimuli [25]. It is reported that shikonin protects H9c2 cells against H/R *via* the PI3K/Akt pathway [26]. Through this pathway, salidroside exerts its protective role in H9C2 cells undergoing oxidative stress injury [27]. In this paper, the protein level of Akt was markedly downregulated following H/R, which was then upregulated by SEV treatment. We believed that the Akt pathway was responsible for the cardioprotective effect of SEV against H/R injury.

Cox-2 is an inducible enzyme, which could be rapidly transcribed and translated by I/R [28]. A relevant study has demonstrated that overexpression of Cox-2 is unfavorable to myocardial I/R injury [29]. Our experimental results illustrated that Cox-2 was upregulated in the H/R group. Notably, overexpression of Cox-2 partially abolished the protective property of SEV on the viability of H9c2 cells undergoing H/R injury. As previously reported, Cox-2 activation is positively correlated to Akt phosphorylation and low survival [30, 31]. Here, the downregulated protein level of p-Akt in H/R-induced cardiomyocytes was partially reversed by SEV treatment. Nevertheless, the reversed trend of the p-Akt level was abolished by overexpression of Cox-2. Cox-2 may negatively affect the protective effect of SEV on cardiomyocyte apoptosis following H/R by inhibiting Akt phosphorylation.

## 5. Conclusions

Sevoflurane protects cardiomyocyte damage following H/R through the Akt pathway, and its protective effect was abolished by overexpression of Cox-2. Our results proposed that inhibition of Cox-2 could assist the protective role of sevoflurane on H/R injury.

## Data Availability

The datasets used and analyzed during the current study are available from the corresponding author on reasonable request.
